# Renewable and high-purity hydrogen from lignocellulosic biomass in a biorefinery approach

**DOI:** 10.1038/s41598-023-50611-5

**Published:** 2024-01-02

**Authors:** Majd Elsaddik, Ange Nzihou, Guo-Hua Delmas, Michel Delmas

**Affiliations:** 1 IMT Mines Albi, RAPSODEE CNRS UMR 5302, Université de Toulouse, Campus Jarlard, 81013 Albi Cedex 09, France; 2 School of Engineering and Applied Science, Princeton University, Princeton, NJ 08544 USA; 3 Andlinger Center for Energy and the Environment, Princeton University, Princeton, NJ 08544 USA; 4 BioEB, 6 Allée des Amazones, 31320 Auzeville-Tolosane, France

**Keywords:** Bioenergy, Hydrogen fuel

## Abstract

Unprecedented efforts are being deployed to develop hydrogen production from bioresources in a circular economy approach, yet their implementation remains scarce. Today’s Challenges are associated with the shortage in the value chain, lack of large-scale production infrastructure, high costs, and low efficiency of current solutions. Herein, we report a hydrogen production route from cellulose pulp, integrating biomass fractionation and gasification in a biorefinery approach. Softwood sawdust undergoes formic acid organosolv treatment to extract cellulose, followed by steam gasification. High-purity hydrogen-rich syngas at a concentration of 56.3 vol% and a yield of 40 g_H2_/kg_cellulose_ was produced. Char gasification offers the advantage of producing free-tar syngas reducing cleaning costs and mitigating downstream issues. A comprehensive assessment of mass and energy balance along the hydrogen value chain revealed an efficiency of 26.5% for hydrogen production, with an energy requirement of 111.1 kWh/kg_H2_. Optimizing solvent recovery and valorization of other constituents as added-value products in a biorefinery approach would further improve the process and entice its industrial takeoff.

## Introduction

Hydrogen (H_2_) is considered a main pillar in the transition for achieving decarbonization. H_2_ produced by renewable sources is projected as a compelling storable, transportable, and utilizable energy vector among environmentally friendly solutions for different applications^1^. The hydrogen demand is expected to increase sixfold to reach 530 Mt_H2_ by 2050, with half of this demand in industry and transport^2^. Over the last decade, strong momentum has been observed in the hydrogen chain value elements ranging from production, transport, storage, endpoint applications, to safety. Green hydrogen and renewables were already expanding, but global energy has paved the way for boosting the uptake of hydrogen.

Biomass can be converted into energy vectors using several routes such as thermochemical and biological processes. However, the feedstock heterogeneity yields a difference in product distribution and properties, complicating the process performance. Lignocellulosic biomass consists of three biopolymers: cellulose, hemicellulose, and lignin. Similar to the concept developed for crude oil refining, lignocellulosic biomass resources can be converted into a spectrum of added-value products and energy carriers^3^. This concept of biorefining is a strategic pillar to develop a circular economy^4^. Furthermore, biomass valorization coupled with intermittent renewable energies may contribute to rural development, thus decentralizing energy production and reducing distribution costs^5^.

So far, the chemical fractionation of biomass has been a key step to develop high-efficient integrated biorefineries. This step relies on the separation of the cellulose by isolating lignin and hemicelluloses. The separation can be achieved through chemical pulping processes used in the paper industry^6^. These processes use dissolving solvents to break lignin intramolecular bonds, as well as lignin-cellulose intermolecular linkages, while preserving cellulose fibers^7,8^. However, these processes use exclusively sulfur-containing solvents which generate atmospheric and effluent emissions. Along with pollution, the presence of sulfur in the products limits their sustainable utilization^9^.

The use of organosolv processes offers the possibility for more efficient utilization of lignocellulosic biomass. In the organosolv process, the lignocellulosic feedstock is treated with low-boiling organic solvents such as alcohols and organic acids namely formic and acetic acid owing to the good solubility of lignin and ease recovery^10^. The operations conditions including solvent-to-biomass ratio (S/L), reaction time, temperature, catalyst and particle size are the main parameters influencing the process efficiency^11^. Higher S/L ratios improve the delignification rate^12^. S/L is typically between 3 and 10. Temperature is one of the most influential parameters, as both the solubility of lignin and the characteristics of the solvent strongly depend on it. The operating temperature ranges from 80 to 220 °C. These sulfur-free treatments fit well with the concept of biorefining as they enable efficient separation of biomass components with minor degradation without generating inhibitor products^13^. Despite their advantages in terms of solvent recovery and mild environmental conditions, the high energy consumption and the high cost of the solvent are the main obstacles for the commercialization of organosolv treatment^3^. To address these challenges, we demonstrate that H_2_-rich syngas production from steam gasification of cellulose pulp in a biorefinery approach could be achieved. Cellulose pulp was obtained from a non-degrading and low-energy consumption organosolv process, labeled as LEEBIO^™^ (Low Energy Extraction of BIOmass). The process uses only formic acid (FA) and operates under mild conditions, < 95 °C and atmospheric pressure. To prevent the degradation of hemicelluloses into furfural, a low water content in formic acid is employed. Thus, the recovery of formic acid can be achieved using low-energy technologies^14,15^.

LEEBIO^™^ treatment relies on the hydrolysis of lignin by the cleavage of the ether linkages to form soluble fragments. FA activity also affects the hydrolysis of hemicelluloses through the breakdown of glycosidic bonds to form sugars. Lignin is linked to hemicelluloses by electrostatic interactions rather than covalent linkages^16^. Therefore, this offers LEEBIO^™^ process the advantage to separate cellulose fibers from lignin and hemicellulose at moderate conditions, 85 °C and 1 atm^17^. The profitability of LEEBIO^™^ process is also advocated by the fact that the extracted lignin and hemicelluloses can be used in industrial sectors without changing the existing technologies or infrastructures. Lignin can be used as a direct drop-in substitute for phenols, polyethylene glycol, and their derivatives in their various applications, such as resoles, novolaks, polyurethanes and epoxy resins^18^. Indeed, Organosolv lignin exhibits superior purity and a lower degree of condensation compared to lignin derived from alternative fractionation methods^19^. Separated hemicelluloses syrups can be valorized into animal feed, C5 sugars and furan derivatives^20^.

In addition to its conventional use in the paper industry, cellulose pulp can be transformed into H_2_-rich syngas. Along with pyrolysis^21^, steam gasification has emerged as one of the main thermochemical routes for the conversion of lignocellulosic feedstocks into hydrogen-rich syngas^22^. Nevertheless, the advancement of biomass gasification relies on effective biochar conversion and addressing challenging undesired products, notably tar. The presence of tar in the syngas can cause various problems in downstream processes, impacting process efficiency and environmental sustainability, hindering the commercial establishment of biomass gasification technologies. To address this issue, diverse strategies target the reduction of tar compounds in biomass gasification gas, classified as primary (within the gasifier) and secondary (downstream) methods. Beyond conventional approaches, novel gasification concepts, such as the two-stage process, provide a distinct method to mitigate tar in biomass gasification. This innovative approach divides the process into distinct units, optimizing each stage to minimize tar formation^23^.

**Figure 1:**
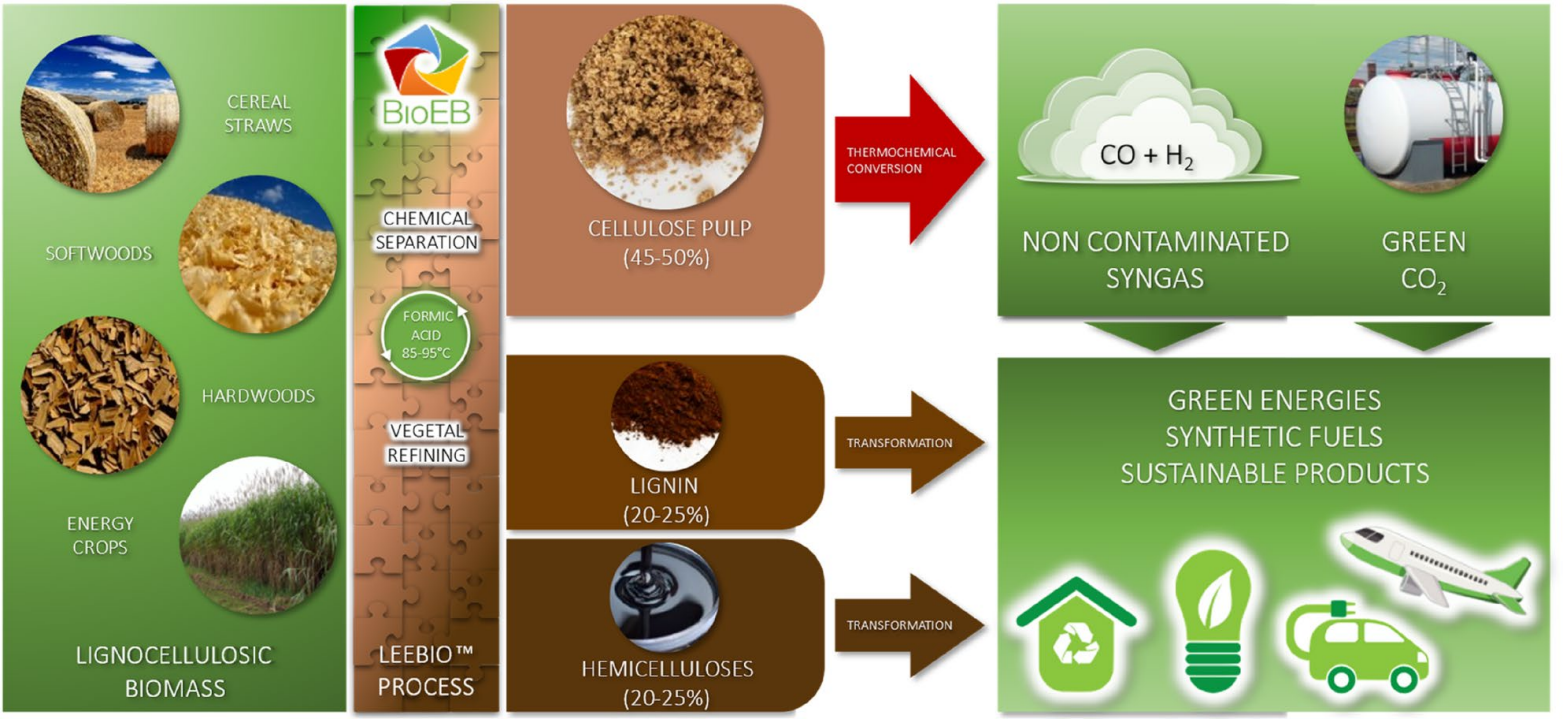
LEEBIOTM technology—cellulose pulp, green energies, fuels, and products from biomass.

The main reactions occurring are the endothermic, requiring heat in the gasifier. The heat required can be autothermal and allothemal^24^. In autothermal or direct gasification, heat is generated by burning part of the feedstock or its pyrolysis products in the gasifier, leading to more efficient energy utilization. However, the resulting product gas has a lower heating value due to the use of nitrogen in the air supply for combustion^25^. Oxygen gasification can be an alternative to improve gas quality (10–18 MJ Nm^−3^) but it is limited by the high oxygen cost and risk^26^. In allothermal gasification, external heat sources are employed, and by using steam as the gasifying agent, high heating value, hydrogen-rich syngas is produced, with a complete carbon conversion^22^. Syngas is a building block to the production of numerous biofuels and biochemicals including H_2_ (Fig. 1). A fraction of the generated hydrogen could be utilized to offset the energy consumption of the process.

In this context, the production of tar-free hydrogen-rich syngas from organosolv cellulose pulp is investigated. Initially, softwood sawdust was fractionated at a laboratory scale to optimize the process in terms of pulping time to reduce energy consumption and to investigate the effect of acid activity on the inorganic composition of the pulp. The process was validated at pilot scale. Subsequently, cellulose was subjected to gasification carried out in a two-stage process starting with pyrolysis followed by char steam gasification. Finally, the process was assessed in terms of H_2_ production efficiency and energy requirement. To this end, the process integrating LEEBIO^™^ treatment and steam gasification was assessed using Aspen Plus software.

## Results

### Biomass fractionation

**Figure 2:**
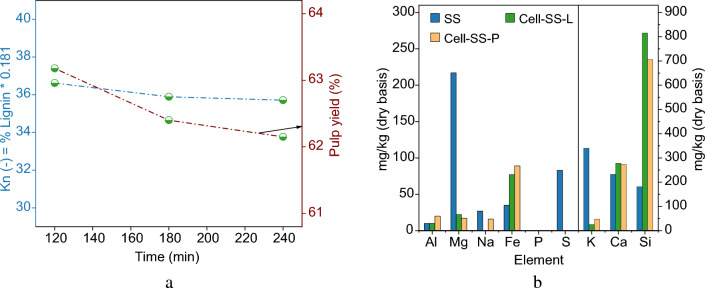
(**a**) Variation of pulp yield and kappa number with pulping time. (**b**) Inorganic composition of SS and the produced pulps; *Cell-SS-L* cellulose pulp obtained from SS at Lab-scale at 180 min of pulping, *Cell-SS-P* cellulose pulp obtained from SS at Pilot-scale.

The softwood sawdust (SS) treatment using LEEBIO^™^ process was optimized at lab scale by applying different pulping times, 120, 180, and 240 min (40 g of SS; solvent-to-biomass (*S/L*) = 5). Pulping time refers to the reaction time between the solvent and lignocellulosic material. Generally, increasing reaction time tends to a higher extent of reaction, but increases energy consumption. The Kappa number (Kn) was determined to give an indication of the content of residual lignin in cellulose pulp. Pulping time influenced residual lignin fraction and pulp yield as shown in Fig. 2a. By extending the pulping time from 120 to 180 min, the lignin fraction residual and pulp yield decreased from 36.6 and 63.2 to 35.9 and 62.4%, respectively. Beyond 180 min, variations in both parameters were minimal. Other studies on organosolv treatment revealed the presence of an optimal pulping time to reach the maximum delignification rate. Indeed, Tu et al. examined the different pulping time ranging from 20 to 180 min, for formic acid organosolv treatement^27^. The maximum delignification rate was predominantly achieved within the initial 80 min. Besides, with extended pulping times, cellulose degradation and lignin recondensation reactions can occur^28^. Consequently, a pulping time of 180 min was selected based on the observed trends in pulp yield and residual lignin content.

The effectiveness of LEEBIO^™^ treatment was assessed by analyzing the chemical composition of cellulose pulp and treated biomass (Table 1). Cellulose was the main component in the pulp. Formic acid (FA) treatment significantly hydrolyzed hemicelluloses by 76.7% and lignin by 33.2% in lab-scale tests. It must be pointed out that the comparison of the obtained results with our studies is limited since the treatment efficiency significantly depends on different parameters including solvent concentration, solvent-to biomass ratio, temperature, and pulping time. Pathak et al. reported 97% hemicellulose, 95% lignin removal and 86% cellulose retention from sugarcane tops at 125 °C for 90 min with 85% FA at a solvent-to-solid ratio of 7.5^29^. Lam et al.^30^ optimized the fractionation of rice straw in FA in terms of acid concentration, temperature and cooking time. A delignification rate of 85% and a pulp yield of 44.4% were obtained by 60 min treatment in 90% FA at 100 °C. The obtained lignin removal rate from SS treatment can mainly be attributed to the structure of the plant cell wall. The latter consists generally of three types of layers, namely the middle lamella (M), the primary wall (P) and the secondary wall (S). Cellulose, lignin and hemicelluloses are differently distributed through these layers^31^. Lignin is highly concentrated in the center corner (CC) and its proportion decreases with increasing distance into the middle lamella^32^. According to literature, during organosolv treatment of straw and hardwood, lignin is removed in the order of S → M → CC^33^. In contrast to other biomass types, softwood delignification can be hindered by the preferential removal of lignin in CC and M regions^34^.

**Table 1:** Pulp yield and the removal rate of lignin and hemicellulose from Softwood sawdust (SS) pulping.

(wt%)	SS	Cell-SS-L^a^	Cell-SS-P^b^
Cellulose	38.5	55.5	60.4
Lignin	31.6	32.5	32.5
Hemicelluloses	29.9	11.1	7.1
Pulp yield		0.63	0.67
Hemicelluloses removal rate		76.7	84.2
Delignification rate		33.2	31.3

^a^Cellulose pulp from SS pulping at Lab-scale.

^b^Cellulose pulp from SS pulping at Pilot-scale.

Under acidic conditions, alkali and alkali-earth metal salts dissolved, reducing K, Na, Mg, and Ca fractions (Fig. 2). P and S concentrations decreased, while Si remained the primary mineral constituent in the pulp^30^, indicating its retention in the epidermic cells in the form of SiO_2_^35^. Despite the acid’s impact on alkali metals, Ca presence was observed in cellulose pulps. Similar observations were found in literature^36^. They reported that part of Ca content can remain in the pulp because of its weak solubility in acid media. Therefore, it can bond with Si to form calcium silicate (SiCa_2_).

To evaluate SS fractionation process, we successfully scaled up the process (scaling factor: 1000) from the lab to pilot scale. The pulp yield, lignin, and hemicellulose removal rates obtained at the pilot scale were comparable to the lab-scale results (Table 1). This confirms that the treatment effectiveness primarily depends on the wood cell structure. Additionally, the mineral composition of the pulp showed similarity between the pilot and lab-scale tests (Fig. 2).

### Steam gasification of cellulose pulp

To investigate the production of high-purity and H_2_-rich syngas from LEEBIO^™^ cellulose pulp (Cellulose-SS-P), we conducted a steam gasification tests in a two-stage approach. This approach has been already established at full scale^37^. The volatiles obtained from pyrolysis are burned to provide heat for steam gasification and pyrolysis unit. Hydrogen-rich syngas is produced from biochar steam gasification. This scheme offers the advantage of generating a tar-free and high purity syngas compared to a single-stage process, hence reducing cleaning costs and avoiding downstream challenges.

Gasification temperature and steam-to-carbon ratio are the main influential operating parameters. Water gas reaction (WGR) (C + H_2_O → CO + H_2_, ΔH =  + 131 kJ/mol) alongside Water–gas shift reaction (WGSR) (CO + H_2_O → CO_2_ + H_2_, ΔH = − 41 kJ/mol) and the Boudouard reaction (C + CO_2_ → 2CO, ΔH =  + 172 kJ/mol) are the main chemical reactions taking place during steam gasification of biochar. Steam-methane reforming (CH_4_ + H_2_O → CO + 3H_2_, ΔH = − 206 kJ/mol) and hydrogasification (C + 2H_2_ → CH_4_, ΔH = − 73 kJ/mol) can equally occur; the effect of both reactions is less significant on hydrogen production and syngas composition. To confirm that gas is the exclusive product derived from biochar gasification and to ensure complete devolatilization, we established the carbon balance of gasification tests (Supplementary Table 2). The data verifies the conversion of carbon in biochar into non-condensable gases, primarily CO, CO_2_, and to a lesser extent, some CH_4_.

To examine the effect of temperature and steam carbon ratio, H_2_/CO, H_2_/CO_2_, CO/CO_2,_ and H_2_/CH_4_ were selected as a scale to assess the gas quality and its production efficiency.

The influence of weight steam to carbon ratio (*S/C*) was studied by varying the steam flow rates (Table 2). As far as the syngas composition is concerned, H_2_ and CO_2_ contents rose slightly while the concentration of CO decreased. The maximum H_2_ fraction reached was 56.3% vol. It is suggested that the increase of *S/C* increased the consumption of CO in the WGSR. Hence, its equilibrium shifts towards the rise of CO_2_ and H_2_ concentration. *S/C* showed a greater effect on CO/CO_2_ which decreased significantly with increasing *S/C*. CO/CO_2_ can be considered as a scale to evaluate the competition between the WGSR and Boudouard reaction which is less dominant in such conditions. The significant decline of 43% in H_2_/CH_4_ could be attributed to the reaction of hydrogasification.

**Table 2:** Influence of steam flow rate on gasification of Cell-SS-P biochar at 950 °C.

Steam flow rate (g/h); 1 h	15	30	45	60
S/C	0.7	1.5	2.3	3.1
Gas production and hydrogen yield				
H_2_ (vol%)	53.4	54.6	56.3	56.3
CO (vol%)	39.0	36.0	31.8	30.4
H_2_/CO	1.4	1.5	1.8	1.9
H_2_/CO_2_	7.4	6.2	5.0	4.6
H_2_/CH_4_	133.6	92.6	75.5	63.4
CO/CO_2_	5.4	4.1	2.8	2.5
H_2_ (g/kg^−1^_cellulose pulp_)	23.0	34.3	40.0	48.1

Besides its presence in the reactants in the WGR, steam flow rate can efficiently enhance the production of hydrogen via the WGSR. Results indicate that hydrogen yield was significantly enhanced with increasing steam amount. The H_2_ yield increased substantially from 109.7 to 190.6 g/kg_biochar_ by changing *S/C* from 0.7 to 3.1. Since the water–gas shift reaction (WGSR) is reversible and can significantly impact the reactivity of steam, benchmarking the optimal *S/C* ratio against the theoretical value is limited. Additionally, the temperature profile within the reactor is non-uniform, with temperatures decreasing as one moves up along the reactor. This non-uniform temperature distribution can influence the kinetics of gas-steam reactions.

It is reasonable to believe that the promotion of biochar gasification could be mainly caused by steam addition. Nevertheless, the results indicate that biochar gasification can be divided into two stages, regarding the fraction of the gas in the product. With the initial increase in *S/C *from 0.7 to 1.5, the gas fraction augmented from 64.9 to 72.9 wt%. This result can be explained by the higher reactivities of carbon-steam reactions, mentioned above. At this stage, the fraction of condensates was relatively insignificant, considering that the steam was converted to gas. When *S/C* was above 1.5, the gas fraction in the product slightly decreased and maintained at 66.6 wt%. Excessive steam resulted in a decrease in steam conversion and a sharp upsurge in the condensate fraction. Similar findings were reported by Yan et al. who suggested that the available biochar was not sufficient to react with all steam injected in the gasifier reactor^38^. Hence, an excessive steam supply can lead to higher energy consumption and operation costs. Similar trends were observed regarding the fractions of recovered biochar. Therefore, optimal biochar gasification occurred within an *S/C* range of 1.5 to 2.3, which is in agreement with the range 2.475–3 reported in the literature for *S/C* ratio^38,39^.

**Figure 3:**
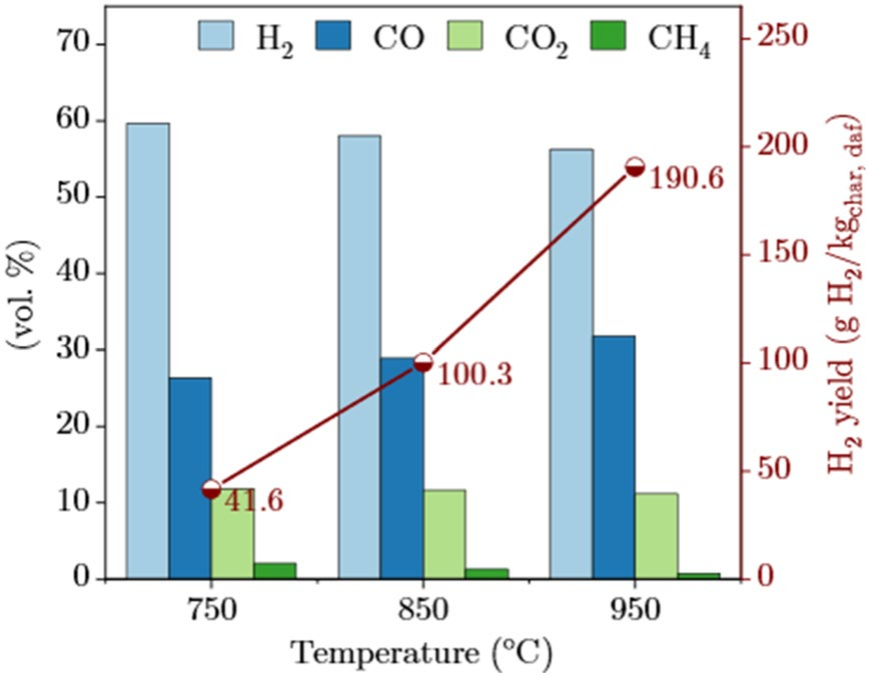
The influence of temperature on cellulose pulp biochar gasification on gas composition and hydrogen yield ($\dot{m}_{steam}$ = 45 g/h).

The temperature is a crucial variable impacting the yields of syngas components, especially H_2_ which is the main product of biochar gasification^40^. The effect of temperature on the gas composition and hydrogen yield is presented in Fig. 3. The evolution of carbon conversion and different gas ratios are listed in Table 2. The maximum H_2_ yield reached 190.6 g/kg_char_ at 950 °C. These results were expected, considering hydrogen is one of the main products of the endothermic reaction of carbon with steam (WGR). This is consistent with what has been found in literature. A full conversion of biochar and a hydrogen yield of 197.8 g/kg char of biochar were achieved by Ning et al.^41^ at 900 °C. Ma et al.^39^ found that with temperature increase from 700 to 900 °C, both H_2_ yield and carbon conversion increased from 28.68 to 83.3 mol/kg char and 49.29 to 90.42%, respectively.

The temperature is a crucial variable impacting the yields of syngas components, especially H_2_ which is the main product of biochar gasification^40^. As shown in Fig. 3, the maximum H_2_ yield reached 190.6 g/kg_char_ at 950 °C. H_2_ fraction dropped gradually with increasing temperature. This is mainly attributed to the decline in reactivity of WSGR which is slightly exothermic and starts to reverse at temperatures between 700 and 800 °C^42,43^. The temperature showed a great impact on the proficiency of syngas components molar ratios. A marked decline in H_2_/CO and a considerable increase in CO/CO_2_ were observed (Table 3). Similar findings were obtained in other studies^44,45^. At higher temperatures, the water–gas shift reaction (WGSR) shifts to the left, leading to a decrease in H_2_ production. It was suggested that high temperatures push left the (WGSR) and the Boudouard reaction becomes more significant when the temperature exceeds 820 °C^45^. The CH_4_ fraction gradually decreased to an average of 0.7 vol% as the temperature increased (Fig. 3). This trend may be attributed to the low reactivity of the hydrogasification reaction and the simultaneous promotion of methane steam reforming. Hence, H_2_/CH_4_ increased by 160% (Table 3). Notably, the WGR, Boudouard reaction, and methane steam reforming reaction play a more prevailing role and the reverse reaction of hydrogasification reaction might have occurred at higher temperatures^38^.

**Table 3:** Carbon conversion and gas ratios in function of temperature and %Si content in the raw material.

		Cell-SS-P	SS
Inorganic indices	K/Si	0.2	4.4
	%Si	61.1	5.5

	T (°C)		
Carbon conversion	750	10.2	22.8
	850	35.4	48.7
	950	74.1	83.1
H_2_/CO	750	2.3	10.3
	850	2.0	4.7
	950	1.8	2.0
H_2_/CO_2_	750	5.0	2.6
	850	5.0	2.7
	950	5.0	4.0
H_2_/CH_4_	750	29.0	39.8
	850	43.9	51.3
	950	75.5	81.4
CO/CO_2_	750	2.2	0.2
	850	2.5	0.5
	950	2.8	2.0

(%Si is determined from the ratio Si/(Total major inorganic elements)).

Notably, steep distinctions were observed in carbon conversion, CO/CO_2_, H_2_/CO, and H_2_/CO_2_ ratios between biochar produced from cellulose pulp and that from raw biomass (Table 3). These differences cannot be linked to the macromolecular composition in terms of cellulose and lignin^46^ but rather to differences in inorganic matter, specifically AEEM and Si, present in the feedstocks. Among AAEM, K is known for its catalytic role^47^ on carbon-steam reaction (WGR) to enhance hydrogen production^48^, as its revealed higher carbon conversion of biochar from SS (Table 3). On the other hand, Si is considered the main inhibitor element^26^. As a result of acid pulping, Si was found to hinder steam gasification of biochar from cellulose pulp at 750 and 850 °C^49^ . As carbon conversion and gas ratios were roughly similar at 950 °C between different biochar samples, the hampering effect of Si effect was milder on the gasification process. The effect of inorganic elements can be expressed by K/Si weight ratio and % Si (Si/total inorganic content).

To provide insights into the inhibiting effect of Si, the variation of gas ratios was considered. It is worth mentioning that despite variations in carbon conversion, H_2_ concentration in the product gas from cellulose pulp biochar was relatively similar to that from raw biochar. This can be attributed to the reactivity of the WGSR, which is not directly influenced by catalytic and inhibiting inorganic species and shifts forward at lower temperatures^50^. H_2_ is generated from the WGR and WGSR while CO_2_ is present in the Boudouard reaction and the WGSR. By considering the H_2_/CO_2_ ratio we can evaluate the competition between WGR/Boudouard. The higher H_2_/CO_2_ of syngas from cellulose pulp biochar, compared to that from other raw biomass, suggests a stronger hampering effect of Si on the carbon steam reaction (WGR). This is supported by the high CO/CO_2_ in the case of cellulose pulp gasification, indicating that CO_2_ is predominantly consumed through the Boudouard reaction to produce CO.

Apart from the effect of Si, the gasification of cellulose pulp biochar at 950 °C demonstrated comparable potential to raw biomass biochar regarding hydrogen yield and concentration. A hydrogen yield of 40 g/kg_cellulose_ and a concentration of 56.3 vol% were achieved. However, it is important to note that the maximum potential hydrogen yield in this study reached only 32% of the theoretical yield of integral biomass gasification, equivalent to 166 g/kg_feedstock_ (Supplementary Table 4). This limitation arises from the use of a multistage gasification scheme, where hydrogen is produced only from biochar steam gasification.

### Proces simulation, heat integration and energy assessment

**Figure 4:**
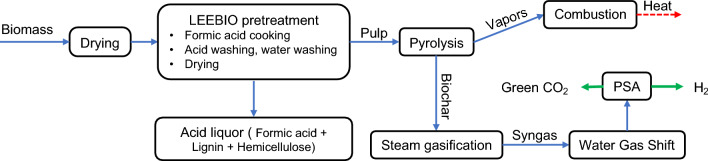
Hydrogen production scheme in a biorefinery approach.

**Figure 5:**
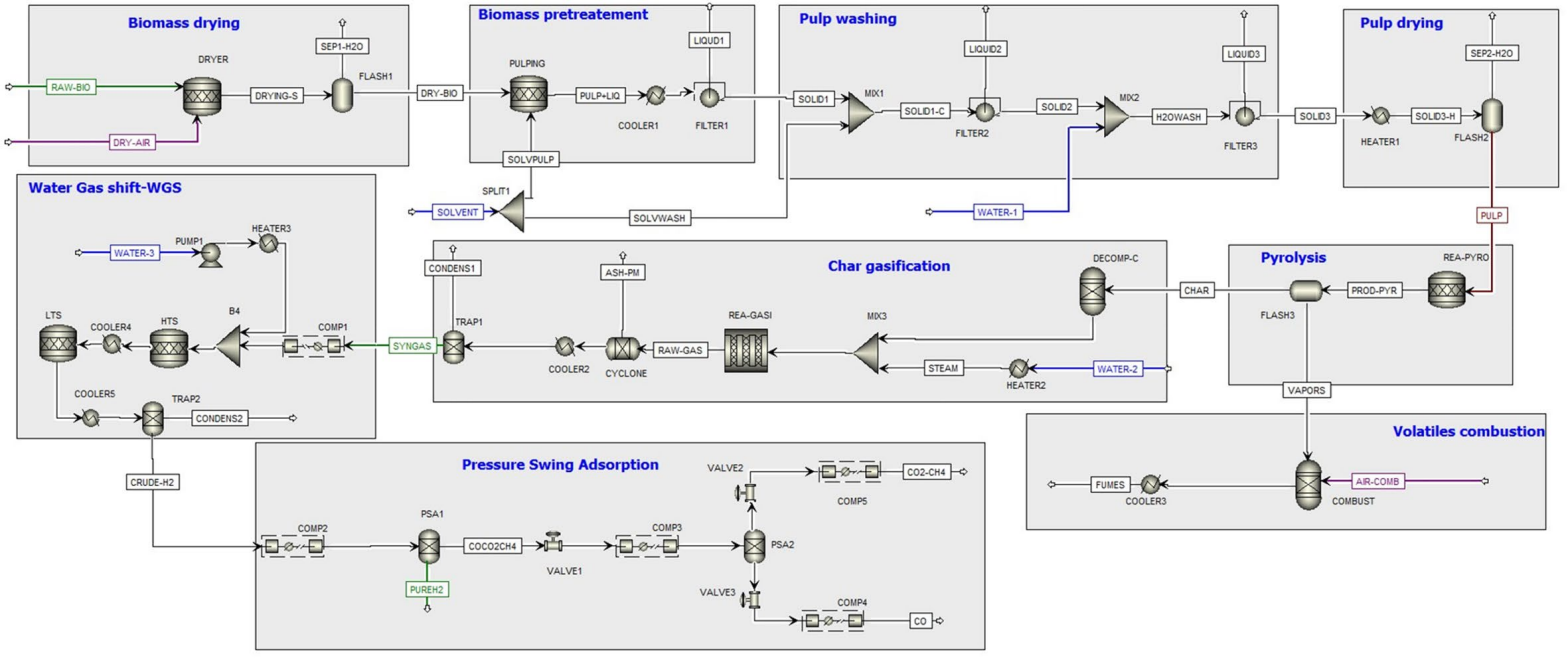
Simulated ASPEN Plus flowsheet for biomass pretreatment and steam gasification of extracted cellulose.

To evaluate H_2_ production from cellulose pulp gasification in an integrated biorefinery approach, we designed an Aspen Plus (V12) model coupling LEEBIO™ treatment and pyrogasification (Fig. 4, flowsheet: Fig. 5).

To assess the model accuracy, a validation was performed by comparing the results of the developed model with the experiments (Supplementary Fig. 6). It can be observed that the model predicted higher CO_2_ concentration at 850 °C. In this set of experiments, CO and CO_2_ showed high error, 12.3 and 10.7%, respectively. Besides, the simulation results exhibited a significant difference from the experimental results for H_2_ yield, with an error of 14.3%. Since the errors related to kinetic constants were minimized, this deviation could be attributed to the inhibiting effect of Si on char gasification, discussed above, were not taken into consideration in the model. The comparison hydrogen molar ratio between the simulation results and the experimental data at 950 °C indicates that the predicted results are in good agreement with experimental data. It can also be noted, that the developed model results followed the trends shown by experimental results of char gasification. The obtained root-mean-square error (RMSE) for H_2_, CO, CO_2_ and CH4 were 1.5, 5.0, 4.0, and 0.4%, respectively. Moreover, the results of the comparisons show a good conformity for hydrogen yield. Using the validated model, the gasification temperature was maintained at 950 °C. The steam input was specified by fixing a carbon conversion of 95%. For hydrogen production, the gasification process was thus coupled to the subsequent steps of water–gas-shift and pressure swing adsorption steps. The operating gasification temperature was maintained at 950 °C. The steam input into the reactor was specified by fixing a carbon conversion of 95%.

The global process includes different steps, down and upstream pyrogasification, requiring energy. These steps are divided between endothermic requiring energy and exothermic releasing energy. On the one hand, the energy needs are identified as thermal under the form of heat and electricity. On the other hand the recoverable energy is the available heat in hot streams. The heating and cooling information of the global process was identified (Supplementary Table 7). As expected, biomass pulping accounted for the highest heat requirement among the energy-demanding units. Overall, the production of 1 kg of H_2_, requires 111.1 kW with 77.9 kW of available recoverable heat. Next, Pinch analysis was applied to determine the energy targets (Supplementary Fig. 7). The electricity consumption was not considered. The cold and hot composite curves for the process were constructed at a minimum temperature approach, ΔT_*min*_ = 50 °C. The results of the analysis are illustrated in Table 4. In regards to energy targets the analysis shows that the hot duty available was mostly recovered, 77.3 from 77.9 kW, indicating the high potential for energy saving. The minimum hot utility requirement (Q_ℎ_) was 27.7 kW, while the cold utility requirement (Q_*c*_) was negligible (0.6 kW). The Pinch was at 25 and 75 °C, for the cold and hot stream, respectively. Decreasing the ΔT_*min*_ towards the threshold value (10 °C) was possible, thus shifting the cold composite curve to left side which would increase the hot utility requirement (Q_ℎ_) and cancel the cold utility requirement (Q_*c*_). Hence, the ΔT_*min*_ was maintained at 50 °C, as Q*c* was negligible.

**Table 4:** Summary of Pinch analysis and energy efficiency results (hydrogen production basis 1 kg/h).

Parameters		Value
Energy balance		
Total heat demand (kW) ①		105
Electrical energy (kW) ②		6.1
Heat available for recovery (kW)		77.9
Pinch analysis (ΔT_min_ = 50 °C)		
Hot temperature pinch (T_h Pinch_) (°C)		75.0
Cold temperature pinch (T_c Pinch_) (°C)		25.0
Total recoverable heat (kW)		77.3
Minimum cold utility, Q_c_ (kW)		0.6
Minimum hot utility, Q_h_ (kW) ③		27.7
Material energy content		
H_2_ energy content (kW) ④		33.6
Dry pulp energy content (kW) ⑤		94.3

	Efficiency (LHV) (%)	Energy requirement (kWh/kg_H2_)
Scenario 1	η_en H2_ with heat integration and without considering pulping (%) = ④/(⑤ + ③ + ② − Q_pulping_ − Q_pulp drying_) = 41.5	72.1
Scenario 2	η_en H2_ with heat integration (%) = ④/(⑤ + ③ + ②) = 26.2	105
Methane reforming^a^	65–75	46 (44–51)
Electrolysis^a^	54–67	55 (50–65)
Coal gasification^a^	45–65	59 (51–74)
Biomass gasification^a^	35–50	72 (69–76)

^a^The efficiency and energy consumption of hydrogen production routes^51,54^.

To obtain more insights into the energy assessment, the energy efficiency of H_2_ production (η_EnH2_) was determined based on the first law of thermodynamics by the apparent thermal efficiency (Eq. 2). Two heat integration scenarios (cases 1 and 2, in Table 4) were considered. In case 1, only pulp pyrogasification process is considered. In this case, an energy efficiency of 42.1%, with an energy requirement of 77.9 kWh/kg_H2_ was obtained. The results were in agreement with biomass gasification processes which typically exhibited energy efficiencies ranging from 35 to 65% and energy requirements of 69 to 76 kWh/kg_H2_^51^. Case 2 illustrates an integrated process of pulp gasification with biomass pretreatment. The energy efficiency for high-purity hydrogen production was determined to be 26.5%, with an energy requirement of 111.1 kWh/kg_H2_. This reveals the energy-intensive nature of the organosolv pretreatment. Further assessment in terms of solvent and by-products recovery are necessary to improve the process efficiency. Thus, hydrogen from integrated biorefineries could potentially complement electrolysis (51%, 50–65 kWh/kg_H2_).

When considering the efficient separation of biomass components, profitability can be estimated using basic assumptions about product prices. The primary source of revenue is derived from lignin. For its application as a phenol substitute, a realistic price range might fall between $1100 and $1300 per tonne. Hemicelluloses are separated in the form of C5 and C6 sugar syrup, and an estimated selling price ranges from $350 to $400 per tonne. This price aligns with the global pricing of sugars like glucose or sucrose.

For cellulose pulp intended for pyrolysis, an estimated selling price falls between $400 and $500 per tonne. This pricing corresponds to the global market for unbleached and unprocessed Kraft pulp. The product distribution from softwood treatment under LEEBIO^™^ is expected to consist of 45–55% cellulose, 20–25% hemicelluloses, and 20–25% lignin. With a price range of $100–150 per tonne of dry raw material, the average selling price of the products falls between $550 and $650.

## Discussion

In this work, we have demonstrated a green hydrogen production approach toward integrated biorefinery. Our approach involves mild organosolv fractionation of biomass to separate the cellulose pulp. This step was performed using formic acid at 85 °C at atmospheric pressure. Interestingly, the organosolv treatment was upscaled from a batch laboratory reactor to a 400 L pilot scale reactor. The resulting cellulose pulp was gasified following a two-stage gasification scheme consisting in a slow pyrolysis followed by the biochar steam gasification to produced a free-tar gas. The molar ratios of syngas components were used to assess the competition between the reactions within the biochar gasification process. At 750 and 850 °C, the steam gasification of cellulose pulp biochar was severely hurdled by the inhibitory effect of Si. Importantly, cellulose pulp shows great potential for high-purity H_2_-rich syngas production at 950 °C. As an example, the obtained H_2_ yield was 40 g/kg_cellulose_ with a concentration of 56.3 vol%. With an overall vision of pure H_2_ production, the global process coupling biomass fraction and steam gasification was simulated and assessed at 26.5% of H_2_ production efficiency with an energy requirement of 111.1 kWh/kg_H2_. The potential to produce H_2_ from cellulose pulp and value-added products from lignin and hemicellulose provide new opportunities to develop closed carbon cycle biorefineries from non-food-related biomass.

## Methods

### Materials and chemicals

Softwood sawdust (SS) sieved under < 5 mm was elected as starting material. Formic acid (CH_2_O_2_, 85%) was purchased from Fischer Chemical.

### Biomass chemical fractionation

#### Lab scale test

To perform chemical fractionation at lab scale, 40 g of dried SS was first loaded in a 0.5 L glass jacketed reactor, followed by 200 g of an aqueous solution of formic acid (FA) to obtain a 5:1 solvent-to-solid ratio. Under medium stirring of 50 rpm (round/min), using a mechanical stirrer with a Teflon anchor, the mixture was heated at 85 °C. Once the temperature was stabilized at 85 °C, the pulping reaction started and maintained for 180 min. After the desired reaction time, the mixture was cooled to room temperature. Then, the mixture was transferred into a Buchner filtration apparatus to separate the raw cellulose from the acid liquor 1. Acid liquor 1 was transferred into a glass beaker. After that, the remaining cellulose on the Buchner apparatus was gently washed with FA (85 wt%), then filtered to remove the acid liquor 2 which was mixed with acid liquor 1 and the whole mixture was kept for further manipulations. Finally, the remaining pulp was washed with warm water (40–50 °C) and filtered until a neutral pH of the filtrate was reached. The pulp was dried in the oven at 60 °C for 12 h. The obtained pulp at the lab scale was analyzed for kappa number (Kn) determination (Supplementary Information).

The pulp yield is calculated by the weight ratio: Y_pulp_ = m_pulp_/m_biomass_ (%). The extraction yield of hemicellulose and lignin was calculated from their fractions in the biomass and the cellulose pulp: Y_i_ = (W_i, biomass_ − Y_pulp_⋅W_i, cellulose pulp_)/W_i, biomass_.

#### Pilot scale test

The ROSENMUND filter/dryer RoLab 0.4 m^2^ was used to perform the pilot scale test (Supplementary Fig. 2). For 40.2 kg of dry biomass, 206 kg of 85 wt% FA was added through the Rolab liquid introduction line to obtain a 5:1 w/w solvent-to-solid ratio. This temperature was maintained at 85 °C for 3 h 30 before cooling the reaction medium (observation of a black coloration). The agitation was then slowed down to 7 rpm for 15 h, while the filtration medium was pressurized to 1.5 bar to filter the liquor. The filtrate was stored in a 200 L plastic drum.

134 kg of FA was introduced to the cellulose retained on the filter in 2 stages. Around 500 L of warm water (60 °C) was next introduced by three times to the Rolab. The mixture was stirred and then filtered with a 120 µm filter cloth.

#### Biomass pretreatment effectiveness

The macromolecular composition of the pulp samples was determined using an analytical procedure. The macromolecular composition of the lignocellulosic materials was determined using a laboratory analytical and gravimetric procedure based on the standards used in the paper industry. The raw biomass and cellulose pulp samples were first crushed at 50 and dried for 12 h at 60 °C, before the determination of cellulose, hemicelluloses, and lignin content. In the next step, extractives were eliminated using an acetone Soxhlet extraction. The lignin content was determined by the sum of soluble and insoluble (Klason) lignin as described in TAPPI standard T222 om-83. The elemental sugars content was measured for the biomass and cellulose pulp samples using ion chromatography after a two-stage sulphuric acid hydrolysis of samples, as described in TAPPI standard T249 cm-80 as detailed in another study^52^. The neutral monosugars obtained were quantified by ion chromatography using a Dionex ICS5000 system.

Emphatically, it must be pointed out that the comparison of the titrimetric analysis (Kappa number Kn) to gravimetric analysis would display some difference since the first is a destructive technique.

### Steam gasification of cellulose pulp biochar

**Figure 6:**
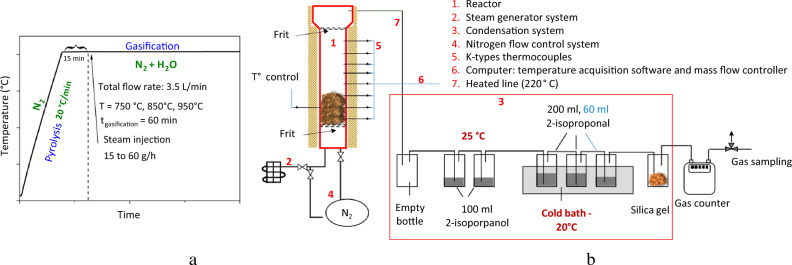
(**a**) Experimental program of pyrolysis and gasification tests. (**b**) Schematic presentation of pyrogasification experimental setup.

Gasification tests of biochar were performed in a semi-continuous fixed bed reactor in a multi-stage process approach as shown in Fig. 6. The process was divided into: (1) devolatilization (or pyrolysis) and (2) biochar gasification. Volatile matter and biochar were produced within the first stage. Volatiles were removed from the reactor and not included in the steam gasification stage.

During the experiment, 90 g of the sample was heated under a nitrogen flow of 3.5 L/min at a heating rate of 20 °C/min to complete the pyrolysis step. After achieving the desired temperatures (750, 850, and 950 °C), the reactor was kept under an inert atmosphere for 15 min. After that, steam was continuously injected (isotherm for 1 h) with nitrogen to make up a mixture including 60 vol% (Supplementary Fig. 3). At the end of the gasification step experiment, the heating was turned off and the atmosphere was switched to nitrogen until the room temperature was reached. Syngas components including H_2_, CO, CO_2_, CH_4_, and N_2_ are analyzed using gas chromatography (micro GC-3000A, Agilent). The remaining char was collected and weighed.

Pyrogasification tests were conducted twice. For each experimental condition, the repeatability was found to be satisfactory, as the calculated standard deviation of the product yield was below 5%.

### Process simulation

#### Conceptual design

Aspen Plus V12 software is employed for process simulation. The elements and methodology of the model are elaborated upon in the Supplementary Information. Overall, the global process involves five linked parts (Fig. 4):

1. *LEEBIO pretreatment: the raw* biomass is firstly dried to reduce moisture content. Dried biomass is then treated with formic acid (FA). From this pretreatment step two streams are obtained: a liquid stream with lignin and hemicellulose dissolved in the solvent, and a solid stream with cellulose which is washed and dried.
2. *Pyrogasification* extracted cellulose undergoes the pyrolysis step to produce volatiles (gas and bio-oil) and biochar at 700 °C. These two products are separated. At this point, biochar is gasified with steam as a gasifying agent to produce hydrogen-rich syngas. The temperature range for the kinetic model employed in the simulation for char gasification was between 800 and 950 °C.
3. *Water–gas-shift reaction* the product syngas is upgraded in a shift reactor for additional hydrogen generation and to convert CO to CO_2_.
4. *H*_*2*_* purification* A pressure swing adsorption (PSA) system to recover and purify the hydrogen present in the upgraded syngas.
5. *Combustion of volatiles from pyrolysis* this step is done in a separated reactor to satisfy the heat demand of the pyrolysis and gasification reactors as well as pretreatment units.

#### Steam gasification model validation

To evaluate the robustness of the model, the root-mean-square error (RMSE) was used to quantify the deviation from experimental results as follows:

1 $${\text{RMSE}}= \sqrt{\frac{\sum_{{\text{i}}}^{{\text{N}}}({{\text{y}}}_{{\text{exp}},{\text{i}}}-{{\text{y}}}_{{\text{exp}},{\text{i}}}{)}^{2}}{{\text{N}}}}$$

where y and N are the mole fraction of syngas species and the count of data point sets,

respectively.

#### Pinch analysis

In this study, Pinch analysis is used to examine the energy integration of the process. This methodology provides a rigorous, structured approach for determining energy targets and optimizing energy saving and usage^53^. The approach is based on thermodynamic principles. At the industrial scale, energy saving can be achieved by heat recovery from hot streams and duties which require cooling and hot steam and duties that need heating. A key tool of Pinch analysis is the graphic of composite curves which determines the minimum energy consumption. It illustrates the profiles of process heat availability (hot composite curve) and heat demands (cold composite curve). The energy targets are determined by overlapping the hot and cold composite curves separated by ΔT_*min*_. The energy targets include the total recoverable heat, minimum hot utility requirement (Q_ℎ_), and minimum cold utility requirement (Q*c*). ΔT_*min*_ refers to the minimum allowable temperature difference. This parameter is selected based on the shape of the composite curves and other factors related to the process.

### Hydrogen efficiency

The energy efficiency of hydrogen production is defined based on the first law of thermodynamics by the apparent thermal efficiency which is applied as follows:

2 $${\eta }_{En H2}= \frac{{E}_{n H2}}{{E}_{n pulp}+ {E}_{n\, demand} }= \frac{{\dot{m}}_{H2} \times {LHV}_{H2}}{{\dot{m}}_{dry\, pulp} \times {LHV}_{pulp}+Q}$$

where, ${\dot{m}}_{H2}$ and ${\dot{m}}_{dry\, pulp}$ are respectively the mass flow rate of produced H_2_ and pulp.

${LHV}_{H2}$ and ${LHV}_{pulp}$ are respectively the low heating value of H_2_ (120.9 MJ/kg) and cellulose pulp; *Q* refers to the process energy input that was considered in two heat integration scenarios (case 1 and 2), as shown in Table 4. 

### Supplementary Information

Supplementary Information.

## Data Availability

All data generated or analyzed during this study are included in this published article and its supplementary information files.
