# Supplementary material for: Renewable and high-purity hydrogen from lignocellulosic biomass in a biorefinery approach
Source: Sci Rep. 2024 Jan 2;14:150. doi: 10.1038/s41598-023-50611-5 (PMC10762170; doi:10.1038/s41598-023-50611-5)
Supplement: Supplementary file 1 — Supplementary Information. [file 41598_2023_50611_MOESM1_ESM.pdf]

Supplementary information

Renewable and high-purity hydrogen from lignocellulosic biomass in a biorefinery approach

Majd Elsaddik[1], Ange Nzihou[1,2,3✉], Guo-Hua Delmas[4] and Michel Delmas[4]

[1] *Université de Toulouse, IMT Mines Albi, RAPSODEE CNRS UMR 5302, Campus Jarlard, F.81013 Albi Cedex 09, France*

[2] *Princeton University, School of Engineering and Applied Science, Princeton, NJ 08544, USA*

[3] *Princeton University, Andlinger Center for Energy and the Environment, Princeton, NJ 08544, USA*

[4] *BioEB, 6 Allee des Amazones, 31320 Auzeville-Tolosane, France*

✉ *e-mail: ange.nzihou@mines-albi.fr; ange.nzihou@princeton.edu*

Supplementary methods

Kappa number

The Kappa number (Kn) indicates the content of residual lignin in cellulose pulp. It is determined through a titrimetric analysis according to Tappi T236 standard method. It is calculated from the amount of potassium permanganate consumed by the lignin in 10 min at room temperature. Uncooked particles of raw biomass were eliminated to guarantee more precise measurements. The analysis was performed three times to ensure the repeatability of the measurements.

For the Kn determination, 1 g of dry cellulose pulp was added to 200 mL of distilled water. First, the solution was mixed using a high-speed mixer and then transferred to an 800 mL beaker under moderate-speed stirring. Next, the residual cellulose pulp on the mixer was rinsed with 200 mL of distilled water; the mixture was then added to the 800 mL beaker.

Under stirring, 50 mL of 0.02 M potassium permanganate solution and 50 mL of 2 M sulfuric acid solution were added. The first reaction thus takes place between lignin, the permanganate, and the acid (Eq. 1) The reaction time was kept at 10 min. After that, 10 mL of KI solution (1N) was added, and the second reaction occurs (Eq. 2). The solution changed from purple to brown color, and 4-5 drops of Starch solution was added which was used as a color indicator. Finally, the mixture was titrated with sodium thiosulphate of 0.2 M to the endpoint (Eq.3). A blank solution, without a sample, was carried out according to the same protocol.

$$Liq + MnO_4^- + 4H^+ \rightarrow \text{Oxidized Lig} + MnO_4^- \text{(Excess)} + MnO_2 + 2H_2O \quad \text{(Eq. 1)}$$

$$2MnO_4^- + 10I^- + 16H^+ \rightarrow 2Mn^{2+} + 5I_2 + 2\,H_2O \quad \text{(Eq. 2)}$$

$$I_2 + 2SO_2O_3^{2-} \rightarrow 2I^- + S_4O_6^{2-} \quad \text{(Eq. 3)}$$

The volume of consumed potassium permanganate solution by lignin is determined following:

$$V = \frac{(b-a)\times M}{0.1} \quad \text{(Eq. 4)}$$

Where a and b are the equivalent volume of sodium thiosulphate solution (mL) used for the determination of the sample and the blank solution, respectively. M is the molarity of sodium thiosulphate solution. The Kappa number Kn is thus determined according to:

$$Kn = V/m * d \quad \text{(Eq. 5)}$$

where m is the weight of the pulp sample and d refers to the correction factor which depends on the quantity of consumed $KMnO_4$. the values of d range from 0.958 to 1.044. The lignin content is calculated as follows:

$$Lignin = Kn * 0.181 \quad \text{(Eq. 6)}$$

Aspen plus model development

1. Methodology
   a. Assumptions

Process simulation was simplified, and the following assumptions were made:

- All units are operated at steady-state, isothermal, and isobaric conditions. Pyrolysis and gasification units are operated at 1 bar
- Drying and devolatilization occur instantly
- Ash are inert; catalytic and inhibiting effects are neglected
- Ideal gas considered
- Heat loss and pressure drops are neglected, temperature and pressure distribution within the units are uniform

   b. Definition of physical property method and operations units

Biomass and its components as well as char and ash are defined as non-conventional solids in this process. Aspen Plus assumes that these components are heterogeneous solids and do not participate in phase equilibrium calculations. HCOALGEN and DGOALIGT library was used to determine the enthalpy and density based on their properties shown in Supplementary Table 1. The MIXCINC stream class was used since both conventional and non-conventional solids are present, but there is no particle size distribution.

2. Aspen flowsheet and model description

Supplementary figure 5 shows the Aspen Plus flowsheet process. Forty-five streams were used in the flowsheet. RAW−BIO, DRY−AIR, SOLVENT, WATER−1, WATER−2, AIR−COMB and WATER−3 were user-defined streams. The flowsheet comprises twenty-nine unit operation blocks, described in Supplementary Table 5. The flow rate of inlet streams in biomass pretreatment steps is determined by a Calculator based on the experimental results. To validate the present model, the experimental results of biochar gasification, a Design−Spec is carried out to ensure production of 90 $g.h^{-1}$ of Cell−Pulp in the PULP stream.

a. Biomass drying

The raw biomass, represented by the stream RAW−BIO, with a moisture content around 39 % is dried with forced air at 70 °C. In this model, drying is carried out in a block DRYER in which moisture is evaporated and controlled by a Calculator to reduce the moisture content to 2 % in the dried biomass. The evaporated moisture is separated from the solid by FLASH1. Aspen plus considers that the molecular weight of a non-conventional solid is 1 g mol−1. Therefore, the drying reaction of biomass is represented by the following equation:

$$\text{Raw Biomass} \rightarrow \frac{1}{M_{H2O}} H_2O \qquad \text{(Eq. 7)}$$

The extent of the reaction in the RStoic is defined by: $\frac{H2O_{in}-H2O_{out}}{1-H2O_{in}}$

b. Biomass drying

In the first step, the outlet stream DRY−BIO of FLASH1 moves into PULPING reactor. The stream SOLVPULP, consisting of a formic acid–water mixture (85 wt.% FA), is added to the organosolv reactor at 5:1 w/w as a solvent-to-wood ratio. The reactor was pressurized to 1.5

bar at 85 °C. During this process, the biomass is fractionated following Eq. 8 . Separated lignin and hemicellulose are not represented in the obtained solid since they are dissolved.

$Dry\ Biomass \rightarrow 0.7\ Cell - Pulp$ (Eq. 8)

c. Solid washing

Following that, the solid is cooled down and filtered by FILTER1 to separate dissolved lignin and hemicellulose. Next, the filtered solid is sent to an acid-washing stage to remove recipitated lignin. This step is represented by MIX1, in which 85 wt.% FA is mixed with the filtered solid at the liquid: solid ratio of 3.4. The mixture is then filtered in FILTER1. Acid washing is followed by water washing (MIX2) and filtration (FILTER2) to clean the solid and remove FA. The required water rate is determined using a Calculator with a water: dried biomass ratio of 12.

d. Drying

Next, washed solid is dried to obtain a final moisture of 2.2%. The step is carried out in HEATER1 to evaporate water and FLASH2 to separate water vapor from the solid.

e. Pyrolysis

The pyrolysis step is simulated using REA-PYRO reactor. Ext-Cell is converted to gases, bio-oil, and biochar. The product distribution information is derived from experimental data. The following assumptions are made on volatile products:

- Gaseous products includes H_2, CO, CO_2, CH_4, and C_2H_4
- Bio-oil consists on water and tars which includes C_6H_6, C_7H_8, C_6H_6O and $C_8H_{10}O_2$

The reactor is operated at 700 °C and at atmospheric pressure; the yield distribution is determined through:

$Cell\ Pulp \rightarrow \frac{1}{M_{H2O}} \text{component}_i$ (Eq. 9)

where the M_i is the molecular weight of a single product; the conversion of each reaction is the yield of $Component_i$. From the REA-PYRO the product stream, PROD-PYR, enters FLASH3 for separating char in the stream SOLID and volatile products in the stream VAPORS.

f. Volatiles combustion

Volatiles obtained from pyrolysis are burned to provide heat for the endothermic steps. As mentioned in the manuscript, this approach is used in the multi-stage gasification process, WoodRoll. The stream VAPORS and AIR−COMB are fed to the COMBUST reactor (Aspen ID: RGibbs). The heat of combustion fumes is recovered to achieve energy integration of the global process (COOLER3).

g. Char gasification

From FLASH3 the stream CHAR, is first fed into DECOMP−C to decompose char into conventional components (Cs, H2 and O2) and ash. The yield of the reactor is calculated according to the elemental composition of char given in Supplementary Table 1. The outlet stream CHAR is then mixed with steam provided by the superheated stream WATER-2. The total input enters the gasifier REA-GASI (Aspen ID: RPlug). In this study, the reactions included in the char gasification process are listed in Supplementary Table 6 and initiated in REA−GASI block. It should be pointed out that the pre-exponential factor of forward WGSR is adjusted. This can be explained by the rapid kinetics of the reaction and the configuration of the lab-scale reactor used in the experimental study. Indeed, the temperature decreases gradually along the reactor which can promote the forward WGSR.

The reactor is about 6 cm in diameter and 60 cm in length and operates at different temperatures, 850 and 950 °C. The outlet gas steam of the gasifier enters CYCLONE for separating ash and residual carbon from the gas stream.

h. Water-gas shift

Afterward, the gas stream is then cooled down to 300 °C and separated from water through TRAP1. Before entering the water-gas shift, pressure the syngas (SYNGAS) to 5 bar. The compression is achieved by a multistage compressor COMP1. The steam input is supplied by pumping water (Water−3) up to 5 bar, then heated to 180 °C. The syngas and steam streams are fed the water-gas shift conditioning section which consists of two reactors, a high-temperature shift (HTS) and a low-temperature shift (LTS) reactor. The WGSR is exothermic and hence a lower temperature enhances CO conversion. At the industrial scale, the HTS generally operates at temperatures between 290 and 440 °C, and the LTS often operates between 160 and 210 °C. Syngas leaving the HTS and LTS shift reactors still contain 2 to 5 % and 0.6 to 1 % CO[1].

Both shift reactors are simulated as stoichiometric reactors (Aspen ID: RStoic) and the WGSR. CO conversion and steam injection flow rate (WATER−3) are adjusted indirectly. This adjustment is carried out using a Design−Spec to specify the residual % vol of CO at the

leaving stream of each reactor. CO concentration in the outlet steam of HTS and LTS is fixed at 4 and 0.8 vol. %, respectively. The CO conversion from the global water-gas-shift system is thus around 80 %. The syngas and steam mixture is cooled down to 35 °C to separate water (TRAP2).

i. Pressure Swing adsorption (PSA)

The target of this stage is to obtain a high-quality hydrogen gas that can be used in subsequent applications. Gas cleaning is achieved by separating hydrogen from other gaseous species present in the syngas. The simulation of the PSA system is simplified by employing ideal separators representing two separation units. The system efficiency and conditions are adopted from literature[2]. The stream CRUDE−H2 obtained from water-gas-shift is compressed at 30 bar and cooled down to 35 °C by a multistage compressor COMP2 to feed the PSA system. Within the first unit (PSA1), high-quality hydrogen (99.99 % purity) is separated with a specified recovery rate of 80 % and exists the PSA bed (PURE−H2) from the bottom stream at the same pressure of the inlet stream. The depressurization and pressurization of the tail gas streams are simulated by VALVE1,2,3 (1 bar) and COMP3,4,5 (30 bar). In the second PSA unit, CO is adsorbed and separated while CO_2 and CH_4 exit from the top stream for their subsequent sequestration.

Supplementary Tables

Supplementary Table 1 | Components of the biomass and the daily wastes

	Proximate analysis (dry basis, wt. %)				Ultimate analysis (dry basis, wt. %)				
	M	VM	FC	A	N	C	H	O*	HHV
SS	39.2	81.5	18.2	0.3	0.1	47.8	6.2	45.9	20.5
Cell-SS	1.7	82.2	17.5	0.3	0.0	48.5	6.1	4.4	19.3
Cell-SS biochar	-	6.1	91.6	2.3	0.0	90.2	1.2	6.3	32.1

M: moisture; VM: volatile matter; FC: fixed carbon; A: ash; HHV unit is MJ.kg $^{-1}$; * by difference

Supplementary Table 2 | Carbon balance

the conversion of carbon in the biochar was calculated following:

$$X_C = m_{carbon\ in\ input\ char} \times \left(1 - \frac{m_{carbon\ in\ recovered\ char}}{m_{carbon\ in\ input\ char}}\right) \quad \text{(Eq. 10)}$$

where the weight of carbon in biochar (g) is $m_{biochar}$ (1-X_{ash}) %C. The weight of carbon in syngas was determined according to:

$$m_{carbon\ in\ syngas} \ \frac{V_{gas}\ (L)\times 1000[CH_4\%+CO\%+CO_2\%+2C_2H_m]\times 12}{22.4} \quad \text{(Eq. 11)}$$

The data are presented in Supplementary Table **2**.

Steam flow rate (g.h^{-1}); 1 hour	15	30	45	60
	Carbon balance (wt. %)			
Carbon in syngas	22.8	26.5	35.4	39.1
Carbon in biochar	75.4	74.2	66.2	60.1
Carbon balance	98.2	101.8	101.7	99.2

Supplementary Table 3 | Influence of steam flow rate on gasification of softwood sawdust (SS) biochar at 950 °C.

Steam flow rate ($g.h^{-1}$); 1 hour	15	30	45	60
S/C	0.8	1.6	2.4	3.2
Gas production and hydrogen yield				
H_2 (vol. %)	53.2	55.3	57.0	57.8
CO (vol. %)	37.6	32.5	28.8	25.8
H_2/CO	1.4	1.7	2.0	2.2
H_2/CO_2	6.3	4.8	4.0	3.7
H_2/CH_4	87.7	82.5	76.8	76.8
CO/CO_2	4.4	2.8	2.0	1.6
H_2 ($g.kg^{-1}{}_{cellulose\ pulp}$)	22.5	36.5	43.1	51.9

Supplementary Table 4 | Theoretical yield of hydrogen from biomass steam gasification (full conversion of CO).

Gasification reaction	$CH_{1.5}O_{0.7}$	+	H_2O	→	CO	+	H_2	g of H_2
($g.mol^{-1}$)	24.7		5.4		28		2.1	85
WGSR	CO	+	H_2O	→	CO_2	+	H_2	
($g.mol^{-1}$)	28		18		44		2	81
			Total g of H_2 per Kg of lignocellulosic material					166

Supplementary Table 5 List of unit operations used the simulation

Aspen ID	Model ID	Description and function
RStoic		Conversion reactor with known stoichiometry
	DRYER	Reduce of moisture content of wet biomass
	PULPING	Biomass fractionation into its main components
	REA−PYRO	Elemental decomposition of fuel and product distribution
	HTS	Simulates the water gas shift reaction at high temperature
	LTS	Simulates the water gas shift reaction at low temperature
RYield		Reactor with specifying reaction yields of each component
	DECOMP−C	Decompose the char into conventional components
RPlug		Plug flow reactor handles rate-based kinetic reactions only
	REA-GASI	Char steam gasification
		Plug flow reactor handles rate-based kinetic reactions only
	REA−GASI	Char steam gasification
RGibbs		Reactor with phase chemical equilibrium and composition
	COMBUST	Combustion of volatiles from pyrolysis
Flash2	FLASH1,2	
	FLASH3	Separation of pyrolysis vapors from biochar
Heater		Used to model: Heaters or coolers
	HEATER1	Pulp heating stream at 70 °C to evaporate water
	HEATER2,3	Water heating to generate steam at 180 °C
	COOLER1	Cooling down pulp and solvent mixture, cold T= 50°C
	COOLER2,3	Remove specified heat duty from gas streams
	COOLER4,5	Lowes the temperature of gas outlet from HTS and LTS
Sep		Separates stream into 2 or more outlet steams
	CYCLONE	Separates solid and ash from the syngas
	TRAP1,2	Separates steam from syngas by condensation
	PSA1,2	Simulates pressure swing adsorption modules
Filter	FILTER1	Separation of liquids from solids
Mixer	MIX1-3	Combines streams into one outlet stream
Mixer	SPLIT1-3	Divides stream into 2 or more outlet streams
Compr		Multistage polytropic compressor with an intercooler between each compression stage (Isentropic Eff.: 0.72)
	COMP1	Raises syngas pressure to 5 bar upstream of WGS
	COMP2,3	Raises syngas pressure to 30 bar upstream of PSA
Pump		Simulate a pump and calculate the outlet pressure
	PUMP1	Raises steam pressure to 5 bar upstream of WGS
Valve	VALVE1,2,3	Rigorous modeling of the pressure drop in control valves

Supplementary Table 6 | Reactions implemented in the Aspen Plus model to model biochar gasification[3,4] (temperature range 750-950°C)

Reactions	Reaction rate (Kmol.m^{-3}.s), Ea (KJ.mol^{-1})
$C + H_2O \Leftrightarrow CO + H_2$	$r_{for} = 15.264 \times T \times \exp\left(-\frac{188.27}{RT}\right)[H_2O][C_s]$
	$r_{inv} = 3.88 \times 10^{-11} \times T^2 \times \exp\left(-\frac{52.54}{RT}\right)[CO]\,[H_2O][C_s]$
$C + CO_2 \Leftrightarrow 2\,CO$	$r_{for} = 15.264 \times T \times \exp\left(-\frac{188.27}{RT}\right)[CO_2][C_s]$
	$r_{inv} = 1.03 \times 10^{-12} \times T^2 \times \exp\left(-\frac{19.65}{RT}\right)[CO][C_s]$
$0.5\,C + H_2 \Leftrightarrow 0.5CH_4$	$r_{for} = 1.37 \times 10^{-5} \times T \times \exp\left(-\frac{97.16}{RT}\right)[H_2][C_s]$
	$r_{inv} = 1.25 \times T^{0.5} \times \exp\left(-\frac{112.89}{RT}\right)[CH_4]^{0.5}$
$CO + H_2O \Leftrightarrow CO_2 + H_2$	$r_{for*} = 6.4 \times 10^8 \times T \times \exp\left(-\frac{304.62}{RT}\right)[CO]^{0.5}[H_2O]$
	$r_{inv} = 6.4 \times 10^6 \times T \times \exp\left(-\frac{326.41}{RT}\right)[H_2]^{0.5}[CO_2]$
$CH_4 + H_2O \Leftrightarrow CO + 3H_2$	$r_{inv} = 3.1 \times 10^3 \times \exp\left(-\frac{124.71}{RT}\right)[CH_4][H_2O]$
	$r_{inv} = 3.56 \times 10^{-6} \times T \times \exp\left(-\frac{124.71}{RT}\right)[H_2]^2[C_s]$
*Adjusted constant (K_0 =7.68*10^7)	

Supplementary Table 7 | Data of energy-consuming units and hot streams (hydrogen production basis 1 Kg.h^{-1}).

Thermal energy consumption	T_c (°C)	T_H (°C)	Q (kW)
Biomass drying	25	70	14.6
Biomass pulping	25	85	32.9
Pulp drying	25	70	14.1
Pyrolysis	70	700	12.2
Biochar gasification	309	950	17.1
Steam superheating	25	180	14.1
Total ❶			105.0
Electricity consumption			Q (kW)
Compressors			6.1
Pump			0.002
Total ❷			6.1
Total ❸ = ❶ + ❷			111.1
Available energy for recovery	T_c (°C)	T_H (°C)	Q (kW)
Heat from pyrolysis volatile combustion	1340	200	66.0
Cooling of syngas from gasification	950	350	6.3
Cooling of syngas from HTS	456	210	2.5
Cooling of syngas from LTS	242	35	3.1
Total			77.9

Supplementary Figures

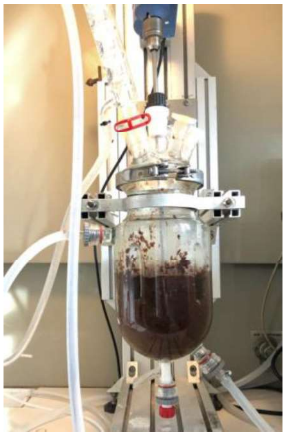

Supplementary Figure 1 | Lab scale fractionation of softwoods sawdust following LEEBIO^{TM}

a b C

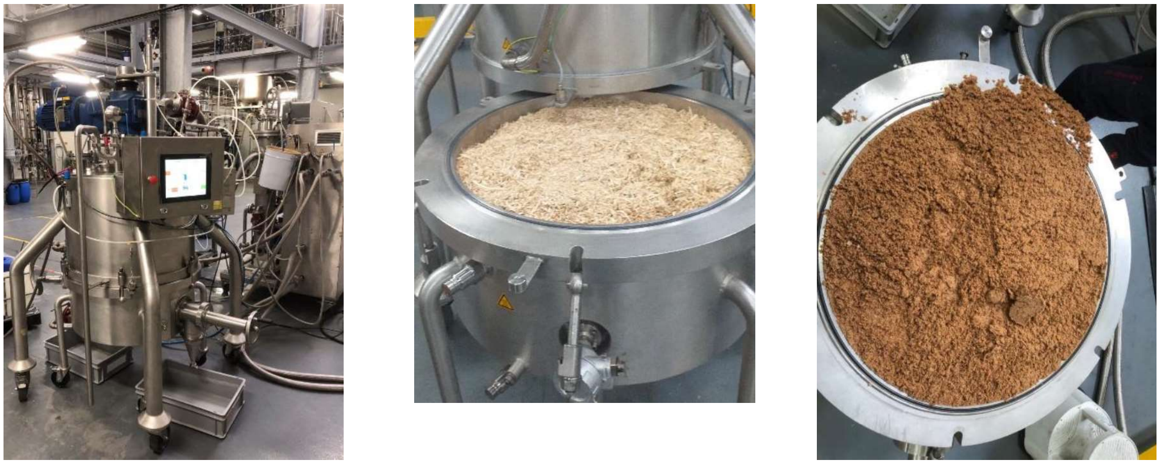

Supplementary Figure 2 | a Softwood pulping following LEEBIO^{TM} using Rolab 0.4 m^2. **b** Sawdsut loading in Rolab 0.4 m^2. **c** Wet cellulose pulp (Cell-SS-P).

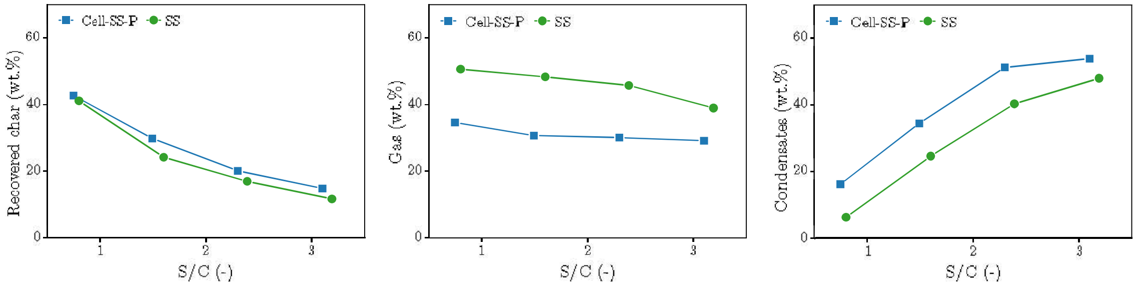

Supplementary Figure 3 | Product yield of different biochar samples as function as S/C at 850 °C.

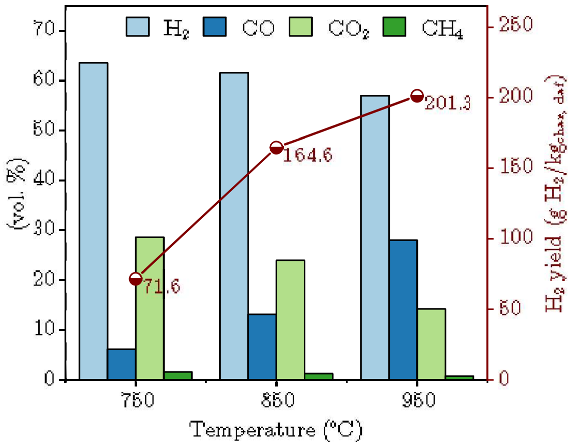

Supplementary Figure 4 | The influence of temperature on softwood sawdust biochar gasification on gas composition and hydrogen yield ($\dot{m}_{steam}$= 45 g.h^{-1}).

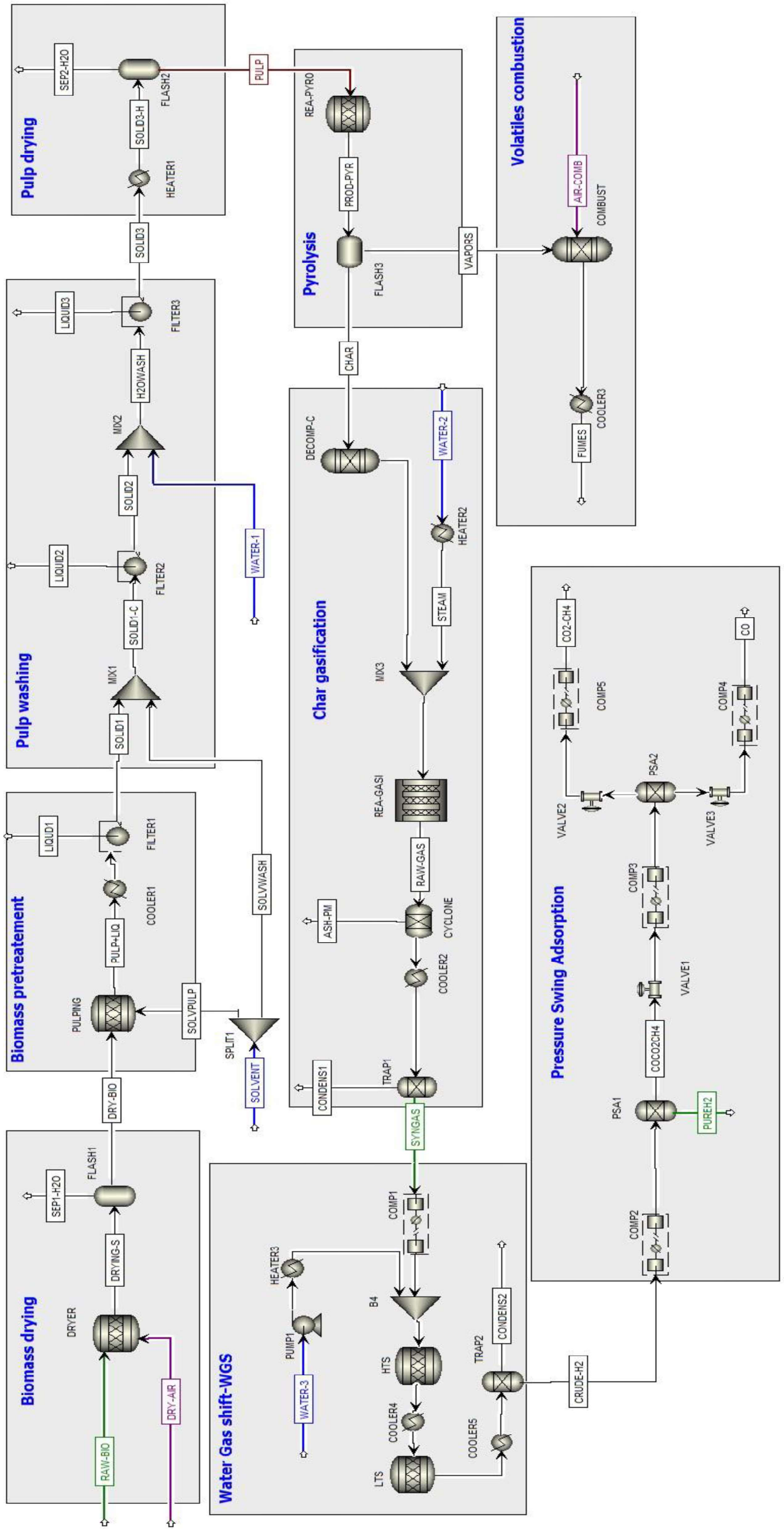

Supplementary Figure 5 ASPEN Plus flowsheet for biomass pretreatment and steam gasification of extracted cellulose

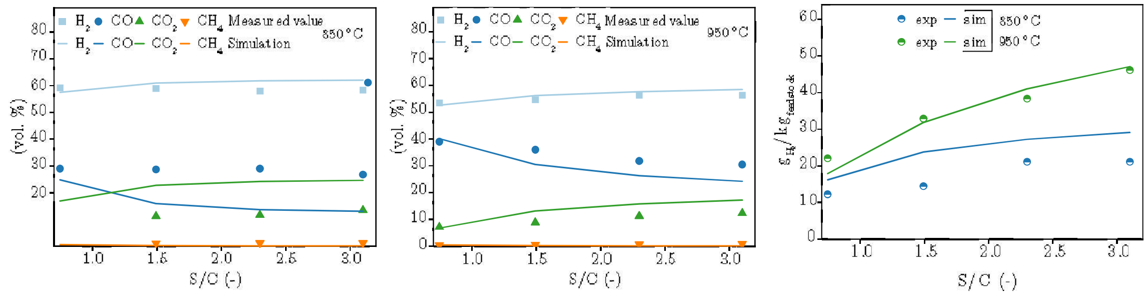

Supplementary Figure 6 | Effect of S/C ratio on gas composition and hydrogen yield at different temperatures: comparison between simulation and experimental data.

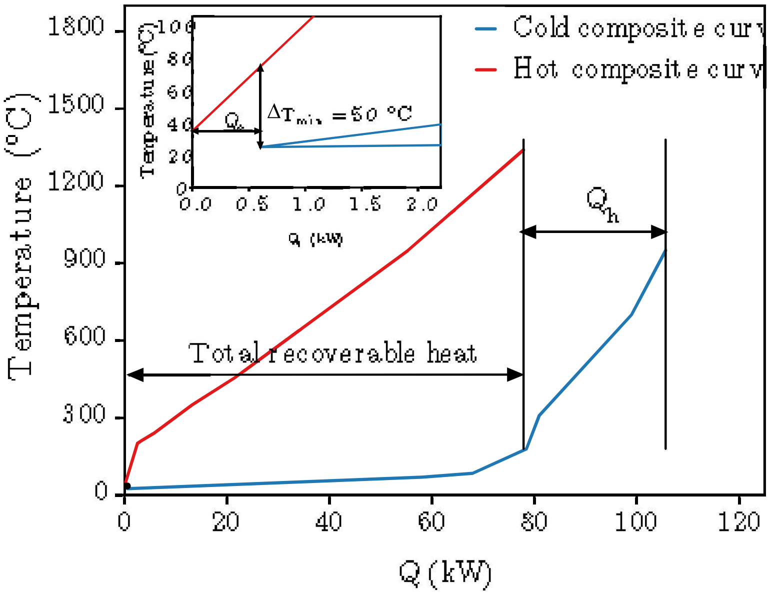

Supplementary Figure 7 | Hot and cold composite curves

References

(1) Linde. CO Shift Conversion.

(2) Gutiérrez Ortiz, F. J.; Serrera, A.; Galera, S.; Ollero, P. Methanol Synthesis from Syngas Obtained by Supercritical Water Reforming of Glycerol. *Fuel* **2013**, *105*, 739–751. https://doi.org/10.1016/j.fuel.2012.09.073.

(3) Eikeland, M. S.; Thapa, R. K.; Halvorsen, B. M. Aspen Plus Simulation of Biomass Gasification with Known Reaction Kinetic; 2015; pp 149–156. https://doi.org/10.3384/ecp15119149.

(4) Solli, K.-A.; Kumar Thapa, R.; Moldestad, B. M. E. Screening of Kinetic Rate Equations for Gasification Simulation Models; 2018; pp 105–112. https://doi.org/10.3384/ecp17142105.
